# A method to audit and score implementation of knowledge translation (KT) interventions in large health regions – an observational pilot study using rectal cancer surgery in Ontario

**DOI:** 10.1186/s12913-020-05353-9

**Published:** 2020-06-05

**Authors:** Marko Simunovic, Christine Fahim, Angela Coates, David Urbach, Craig Earle, Vanja Grubac, Melissa Brouwers, Mary Ann O’Brien, Nancy Baxter

**Affiliations:** 1 Department of Surgery, Faculty of Health Sciences, McMaster University, Hamilton, ON Canada; 2 Department of Health Research Methods, Evidence, and Impact, Faculty of Health Sciences, McMaster University, Hamilton, ON Canada; 3 Department of Oncology, Faculty of Health Sciences, McMaster University, Hamilton, ON Canada; 4 Escarpment Cancer Research Institute, Hamilton Health Sciences and McMaster University, Hamilton, ON Canada; 5 Department of Surgical Oncology, Juravinski Cancer Centre, Hamilton, ON Canada; 6 Department of Health Policy and Management, Johns Hopkins Bloomberg School of Public Health, Baltimore, MD USA; 7 Department of Surgery, University of Toronto, Toronto, ON Canada; 8 Department of Medicine, University of Toronto, Toronto, ON Canada; 9 Faculty of Medicine, School of Epidemiology and Public Health, University of Ottawa, Ottawa, ON Canada; 10 Department of Family & Community Medicine, University of Toronto, Toronto, ON Canada

**Keywords:** Rectal cancer surgery, KT interventions, Region-level KT signature scores

## Abstract

**Background:**

Across Ontario, since the year 2006 various knowledge translation (KT) interventions designed to improve the quality of rectal cancer surgery have been implemented by the provincial cancer agency or by individual researchers. Ontario is divided administratively into 14 health regions. We piloted a method to audit and score for each region of the province the KT interventions implemented to improve the quality of rectal cancer surgery.

**Methods:**

We interviewed stakeholders to audit KT interventions used in respective regions over years 2006 to 2014. Results were summarized into narrative and visual forms. Using a modified Delphi approach, KT experts reviewed these data and then, for each region, scored implementation of KT interventions using a 20-item KT Signature Assessment Tool. Scores could range from 20 to 100 with higher scores commensurate with greater KT intervention implementation.

**Results:**

There were thirty interviews. KT experts produced scores for each region that were bimodally distributed, with an average score for 2 regions of 78 (range 73–83) and for 12 regions of 30.5 (range 22–38).

**Conclusion:**

Our methods efficiently identified two groups with similar KT Signature scores. Two regions had relatively high scores reflecting numerous KT interventions and the use of sustained iterative approaches in addition to those encouraged by the provincial cancer agency, while 12 regions had relatively low scores reflecting minimal activities outside of those encouraged by the provincial cancer agency. These groupings will be used for future comparative quantitative analyses to help determine if higher KT signature scores correlate with improved measures for quality of rectal cancer surgery.

## Contributions to the literature

- Pilot a method to audit and score region-level KT intervention implementation for a specific clinical target
- Demonstrate the complexities of summarizing region-level KT intervention implementation

## Background

Knowledge translation (KT) interventions in health care are meant to facilitate the implementation of evidence-based practice and help close quality gaps [1]. Examples of KT interventions include guidelines, audit and feedback, and use of opinion leaders. Stakeholders have suggested that KT intervention effectiveness may be enhanced through the use of ‘integrated knowledge translation’ (iKT); the use of theory to plan, implement and evaluate any KT strategy; and, sustained iterative approaches that allow KT efforts to be modified as barriers to practice change are recognized [2–5]. With iKT, the target subjects of an evidence-based intervention (e.g., front-line surgeons) are involved in all aspects of the research initiative including design, implementation and evaluation [2, 3]. The Knowledge-to-Action (KTA) Cycle is informed by key behavioural theories (e.g., social theory) that may drive health care worker behaviour and reinforces the importance of an iterative sustained effort to close quality gaps [4, 5].

The province of Ontario, Canada (population 14 million) is divided into 14 health regions [6]. Cancer Care Ontario, the governing body responsible for cancer care across the province, has used various KT interventions to improve the quality of care received by patients diagnosed with cancer, including patients undergoing rectal cancer surgery. These include use of guidelines, communities of practice, diagnostic assessment programs, and multidisciplinary cancer conferences [7–12]. The intention of these latter three interventions, respectively, is to have surgeons work together in a region to develop methods of optimizing care; to facilitate the timely and appropriate testing and treatment of people with cancer; and, to ensure that patients receive coordinated treatment recommendations from a range of specialists. As well, Cancer Care Ontario routinely reports on wait times for cancer surgery and occasionally executes limited audit and feedback to health region administrators (e.g., number of lymph nodes counted in pathology specimens). Of note, these interventions are delivered or encouraged in a top down manner; Cancer Care Ontario administrators have no mechanism to force surgeon engagement with any intervention, nor has there been an effort to evaluate the impact on patient care of these interventions.

There are reports of stakeholders in some Ontario regions engaging in KT activities in addition to those encouraged by Cancer Care Ontario. For example, in year 2006, the sustained iterative Quality Improvement in Colorectal Cancer strategy was initiated in one region of Ontario with a population of 1.4 million [13]. The strategy incorporated iKT principles and was informed by the KTA Cycle. Briefly, front-line surgeons co-designed all aspects of the strategy, including the selection of quality markers for assessment and KT interventions to optimize marker scores. There are published reports of related efforts in at least one other region [14].

Despite the considerable resources involved in such KT activities, we could find no method in the literature to summarize and score region-level KT intervention(s) implementation. We piloted a method to audit region-level KT activities implemented to improve the quality of rectal cancer surgery. Results were then used to ascribe a ‘KT signature score’ for rectal cancer surgery to each of the 14 Ontario regions. Our intention was for such scores to reflect the breadth of KT interventions (e.g., audit and feedback) and approaches to intervention implementation (e.g., sustained, iterative) used in each region over a set time period. Regions with similar scores will be grouped for future comparative quantitative analyses to investigate if higher KT signature scores correlate with improved measures of quality for rectal cancer surgery. Methods and findings should be relevant to other areas of health care.

## Methods

### Study setting and design

**Fig. 1:**
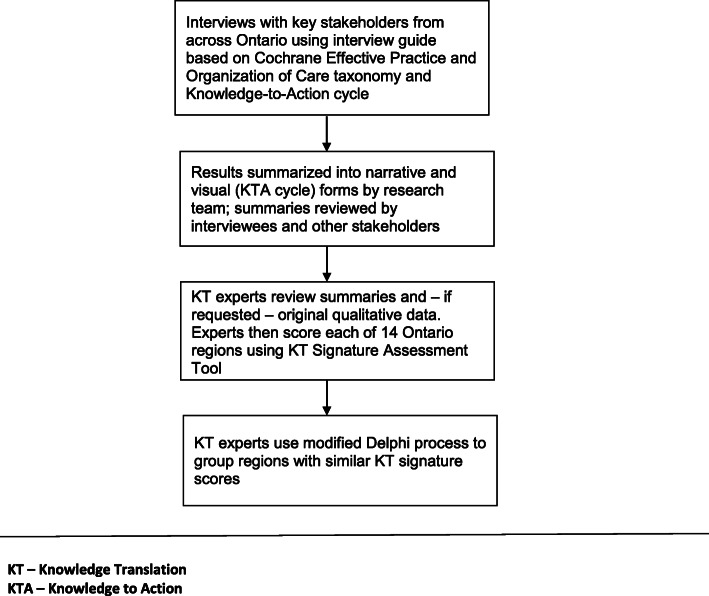
Methods Flow Diagram

This study took place in Ontario, Canada. The aim of our study was to ascribe to each of the 14 Ontario health regions a ‘KT signature score’ related to the types of KT interventions and approaches to intervention implementation used in each region to improve the quality of rectal cancer surgery. We used interviews with key stakeholders to obtain data on region-level KT interventions related to rectal cancer surgery that occurred over years 2006 to 2014. Our audit started at year 2006 since this was the year when Cancer Care Ontario became more active in attempting to influence the quality of rectal cancer surgery through KT interventions. We summarized results into narrative and visual forms. KT experts then scored these summaries using a KT Signature Assessment Tool and grouped regions with similar scores. (See Fig. 1 –Methods Flow Diagram).

### Design of Interview Guide

A 25-page interview guide helped identify if an intervention did or did not occur and processes of intervention implementation. (Additional File 1) The Cochrane Effective Practice and Organization of Care taxonomy outlined an exhaustive list of KT interventions that may have been used including: education materials (e.g., guidelines), education meetings, audit and feedback, practice demonstrations, education outreach or detailing, reminders, and, tailoring interventions [15]. Activities potentially provided though Cancer Care Ontario but not specifically listed in the Cochrane taxonomy such as communities of practice, diagnostic assessment programs, and multidisciplinary cancer conferences were also included. They were included in the interview guide since the goal of these activities is to improve relevant care consistent with optimal current standards, the presumed mechanism of action is the improvement of knowledge among clinicians, and, effectiveness is contingent on local clinician participation. Positive responses were probed further to understand the processes of intervention implementation. Probes considered the following: was the activity selected by an individual or group; what body did such individuals or groups represent; were interventions selected to address specific quality gaps; were interventions delivered at the individual surgeon, hospital or region level; and, how was intervention success evaluated? There was special interest in identifying surgeon-led iKT targeting region-level performance, and, evidence of sustained iterative approaches (e.g., data exercises that were repeated through time and not simply one-off evaluations). These latter concepts were considered reflective of more progressive and effective KT approaches.

### Participants

The Surgical Oncology Program at Cancer Care Ontario assigns a surgical oncology lead and a colorectal cancer surgery lead for each of the 14 Ontario health regions. These leads were invited to participate under the premise that they were the most likely surgeons to be familiar with rectal cancer surgery KT initiatives in their respective region. In addition, heads of general surgery at high volume hospitals (i.e., performed > 10 rectal or rectosigmoid cancer procedures per year) were approached for interviews. Snowball sampling was used to identify other key informants well positioned to provide relevant information [16]. Interviewees received no compensation for participation.

### Data collection and organisation

In advance of interviews, participants received an introductory package that included the purpose of the study and a summary of the interview guide. The summary listed pre-identified KT interventions and relevant processes of intervention selection. A single research coordinator conducted telephone interviews. Following participant consent, the interviews were recorded and transcribed verbatim.

For each region the research team summarized data on implemented KT interventions and processes of implementation into two forms. First, a narrative form summarized the following: 1) KT activities at the provincial, region and individual hospital level, with provincial efforts being common to all regions; 2) how quality gaps were identified and how interventions were selected; and, 3) the KT interventions implemented over the years in the region, with further detail on processes of implementation for each intervention. Second, a KTA cycle was populated where appropriate with specific interventions and processes. For member-checking, narrative summaries and KTA Cycles for individual regions were mailed to interview participants for review and feedback. To further establish comprehensive data gathering, respective region narrative summaries and KTA Cycles were sent to other respective regional stakeholders including Cancer Care Ontario Regional Vice Presidents, Cancer Care Ontario Surgical Oncology and Colorectal Surgery leads (to the small number of such individuals who did not participate in interviews), and Chiefs of Surgery at all hospitals in each region. Covering letters emphasized the importance of stakeholders reviewing summarized region processes and KT activities, since these would be used to assign a KT signature to each region for future quantitative analyses. Feedback and corrections were encouraged. Small clarifications were received from two regions and incorporated into final narrative summaries and KTA cycles.

### Outcomes and analysis

**Fig. 2:**
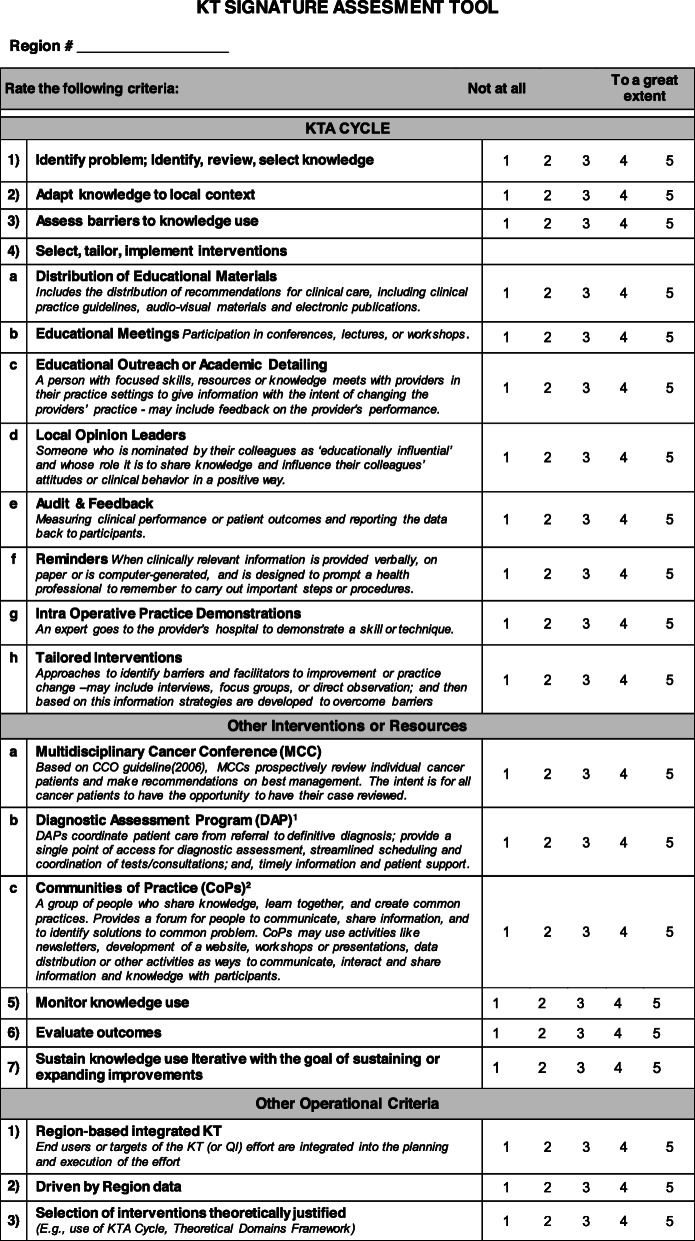
KT Signature Assessment Tool

The primary outcome for this study was a ‘KT signature score’ ascribed to each of the 14 Ontario health regions. The intention was for such scores to reflect the breadth of and approaches to KT intervention implementation meant to improve a region’s quality of rectal cancer surgery. The study team could find no instruments to score KT intervention implementation across a large region and over an extended period of time in any clinical area; or even any articles attempting to do this using either qualitative or quantitative methods. Therefore, we devised a practical KT Signature Assessment Tool. (See Fig. 2) This tool listed 20 items corresponding to processes of implementation (e.g., the use of region-level data to identify quality gaps) and specific KT interventions (e.g., guidelines). Four experts in KT reviewed the tool and provided feedback on its design prior to use. For this initial attempt at assigning KT signature scores, apriori it was decided that each of the resulting 20 items would be scored on a Likert scale from 1 to 5, where 1 and 5 represented the item or process used ‘not at all’ or ‘to a great extent’, respectively. The maximum and minimum score for each region was therefore 100 (20 items × 5 = 100) and 20, respectively.

### Assigning a KT signature score to regions

The above four KT experts also participated in a modified Delphi process to assign KT signature scores to individual regions using our collected data [17, 18]. Experts were first provided with the study objectives and methods, a copy of the final KT Signature Assessment Tool, and, the region summaries (narrative and KTA Cycle). Face-to-face meetings were then arranged. Meetings began with a study overview, and then presentation of each region’s narrative and KTA cycle summary. Primary data were also available for direct review. Following each presentation, raters independently scored intervention implementation using the KT Signature Assessment Tool. It was decided a priori that for each region scores from raters would be averaged. Regions were thus rank-ordered according to these average scores. A priori it was also decided that regions with similar ‘KT Signature’ scores would be placed into groups for potential future quantitative analyses. Experts discussed average scores and the region summaries to formulate region groupings. Consensus for groupings was reached and then re-visited and re-confirmed through post-meeting email.

## Results

**Fig. 3:**
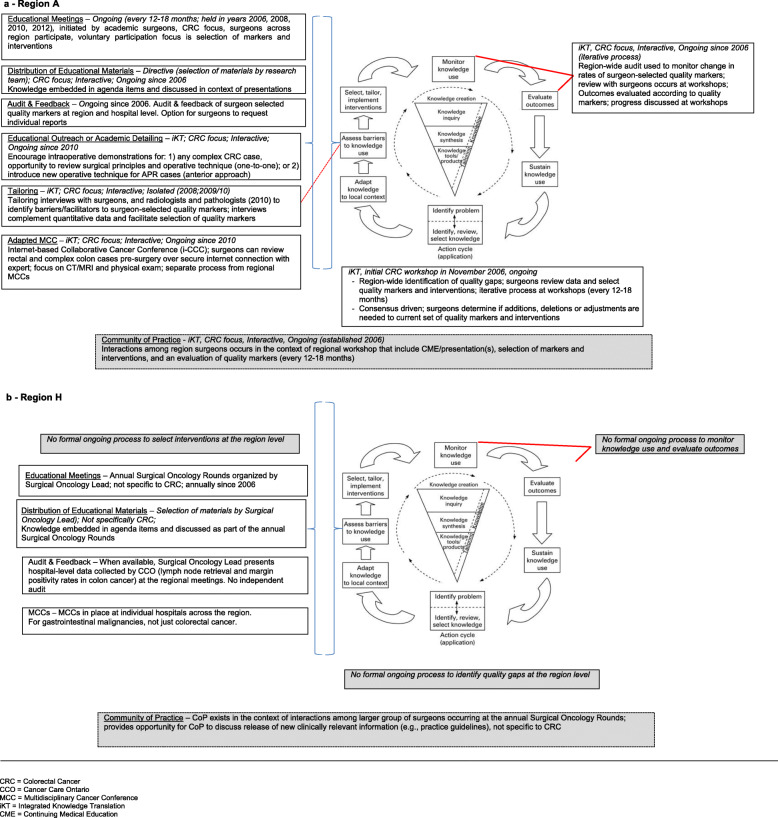
KTA cycles for Regions A and H - KT activities related to rectal cancer surgery in years 2006–2014

Interviews were held between January 2014–March 2015. Two to four interviews were completed per region, for a total of 30 interviews. Only two individuals declined to be interviewed. The average time for interviews was 24.5 min (range 13–45 min). For illustrative purposes, Figure 3a and b presents KTA cycle summaries for region-A and region-H, respectively. Of note, qualitative data including narrative and visual summaries for each region are not presented in this paper.

### Ascribing region-level KT signature scores

The KT Signature scores from our four raters for each region are presented in Table 1. Individual rater scores ranged from 22 to 91, with an overall mean quality signature score of 37 for the 14 regions. Regions were rank-ordered by score. There was a bimodal distribution of scores. In follow-up email communication, experts achieved consensus on two KT Signature groups – ‘KT Signature I’ consisting of regions A and B, and ‘KT Signature II’ consisting of the remaining 12 regions. A mean score of 78 for region-A (83, rank order 1) and region-B (73, rank order 2) was much higher than the scores of the remaining regions (mean score 30.5, range 22–38).

**Table 1:** KT Signature Assessment Tool Scores among 14 Ontario Regions for years 2006–2014

Region	Raters				Points range	Mean score	Rank^a^
	A	B	C	D			
**Region-**A	**91**	**69**	**79**	**91**	**69–91**	**82**	**1**
**Region-**B	**74**	**76**	**82**	**60**	**60–82**	**73**	**2**
**Region-**C	35	39	34	44	34–44	38	3
**Region-**D	36	38	38	38	36–38	38	3
**Region-**E	32	37	45	35	32–45	37	5
**Region-**F	34	34	30	41	30–41	35	6
**Region-**G	35	32	34	36	32–36	34	7
**Region-**H	30	28	32	31	28–31	30	8
**Region-**I	31	30	30	28	28–32	30	8
**Region-**J	29	28	27	27	27–29	28	10
**Region-**K	27	25	24	25	24–27	25	11
**Region-**L	25	25	25	23	23–25	25	11
**Region-**M	25	22	25	23	22–25	24	13
**Region-**N	23	22	22	22	22–23	22	14

^a^Letters are anonymized

Related to rectal cancer surgery, regions A and B were the only regions which utilized formal processes at the region level to identify quality gaps, select KT interventions, monitor knowledge use, evaluate outcomes, and, repeat such activities in an iterative fashion. These regions both executed interventions such as iterative audit and feedback that were in addition to those encouraged by Cancer Care Ontario. As well, both ‘KT Signature I’ regions had surgeon champions initiating KT efforts that attempted to engage all surgeons across the region in colorectal cancer improvement efforts.

In ‘KT Signature II’ regions interventions (e.g., multidisciplinary cancer conferences) were not initiated by front-line surgeons (i.e., iKT was not used) and there was no use of data in an iterative sustained fashion to evaluate progress (i.e., did not use some form of the KTA cycle). In KT Signature II regions, community of practice events were ostensibly limited to continuing education meetings, which may or may not have had a component focusing on colorectal cancer surgery, and may or may not have occurred annually. In one ‘KT Signature II’ region during the 9 years under review there was a single episode of audit and feedback initiated by a regional surgeon champion relevant to colorectal cancer surgery. This involved the collection and reporting of data from some but not all region hospitals.

## Discussion

For each of the 14 Ontario health regions from 2006 to 2014, interviews with key stakeholders provided data for an audit of KT interventions and approaches to intervention implementation used to improve the quality of rectal cancer surgery. KT experts then reviewed the collected data and scored each region using a KT Signature Assessment Tool. Scores resulted in the regions being divided into two groups. The two ‘KT Signature I’ regions used numerous interventions in an iterative fashion and with surgeon leadership and engagement of front-line surgeons. The 12 ‘KT Signature II’ regions showed minimal evidence of initiating region-level interventions outside of interventions encouraged by Cancer Care Ontario (e.g., annual region meetings that may or may not have included lectures on colorectal cancer). Prior to this study we were aware that the two KT Signature I regions had published articles on the implementation of rectal cancer surgery KT interventions in addition to those championed by Cancer Care Ontario [13, 14]. Our results suggest that outside of these two regions, none of the other 12 Ontario regions implemented similarly intense KT strategies.

The two regions that scored highest on the KT Signature Tool had numerous common features. Quality improvement activities were initiated from academic hospitals by clinician-researchers with expertise in KT and quality improvement. From the beginning efforts explicitly attempted to engage all surgeons providing rectal cancer care in the respective region, and both used region-level data in an iterative fashion to pursue improvement. Of interest, one region encouraged the centralization of rectal cancer surgery to academic sites, while this was not a priority of the second region. It is not guaranteed that the KT efforts in the two ‘KT Signature I’ regions have led to markedly improved patient process or outcome measures versus results in the remaining 12 ‘KT Signature II’ regions. It may be that Cancer Care Ontario efforts improved patient measures across the province, rendering redundant the additional KT activities implemented in the two ‘KT Signature I’ regions. Our group plans to compare key process and outcome measures for rectal cancer surgery in ‘KT Signature I’ versus ‘KT Signature II’ regions. These planned quantitative analyses may provide further validation for our method of ascribing a KT signature to large regions. These analyses should also provide insights on the role of iKT and KT theory on region-level quality of rectal cancer surgery, since these progressive methods appeared to have been used only in two of the 14 Ontario health regions.

Despite the considerable resources deployed in many jurisdictions to implement KT interventions, we could find no method in the literature to audit and score the implementation of region-level KT interventions. This is surprising. Health care costs account for a major portion of government budgets and occupy a great deal of policy attention, including in Ontario. Understanding improvement efforts occurring – or not occurring - among front-line clinicians in a given region should be of interest to stakeholders, and could even inform subsequent government policy. Cancer Care Ontario is charged with improving the quality of cancer care in Ontario and is recognized as an international leader in this area [19]. Cancer Care Ontario has an active agenda of KT-like interventions and policy initiatives designed to improve cancer services, including for patients with colorectal cancer. Yet engagement with interventions by front-line clinicians is not mandatory; nor are there ongoing evaluations of the impact of implemented interventions or strategies, or evaluations of clinician engagement with such interventions.

Ideally, individual surgeons or groups of surgeons in a hospital would pro-actively pursue KT improvement projects. But individual surgeons have limited time and expertise to pursue KT projects. It is also likely most surgeons are confident their quality of provided care is optimal; and any improvements in care will occur incrementally through new treatments introduced via journal articles or continuing education meetings. But gaps in care may be difficult to discern in a busy surgical practice, especially when rates of catastrophic complications are low and can be - correctly or incorrectly - attributed to patient factors or chance. We suggest that deliberate and effective methods are needed to ensure ongoing improvement at the population level in all fields of health care. What those methods should be has not been clearly established. Validation of our methods and results, and the ability to ascribe a KT signature to regions in specific areas of clinical care, would be an important contribution to efforts evaluating the impact of KT strategies at a population level.

There are potential weaknesses with this study. First, the method of ascribing a KT signature implementation score to a region has not been validated, including the use of focused interviews and scoring collected data by KT experts using our KT Signature Assessment Tool. However, the KT Signature Assessment Tool did have some face validity since it was put together with the input of KT experts, and, was based on the Cochrane Effective Practice and Organization of Care taxonomy of interventions and the KTA cycle; the former is accepted as an exhaustive list of KT interventions and the latter is accepted as a key theoretical construct for the implementation of KT interventions. As well, KT experts consistently produced a bimodal distribution of KT Signature Scores - higher scores for ‘KT Signature I’ versus ‘KT Signature II’ regions. Regardless, this method of summarizing and scoring KT interventions implemented at a region level should be considered a pilot. Stakeholders from other regions are encouraged to replicate, evaluate, and refine our methods. As well, we also plan to use the identified KT signature groupings for future quantitative analyses – this may provide construct validity for our methods. Second, we scored numerous KT interventions and processes delivered over a number of years. This precluded the use of a checklist approach to describe content for each and every intervention [20]. But our KT experts were aware of concepts such as the presumed superiority of iKT and iterative, sustained efforts. We are confident such relevant factors were integrated into KT Signature Tool scores. Regardless, we again emphasize the need to further validate our methods. Third, it is possible that in a given region, interviews did not identify all relevant KT interventions (i.e., recall bias). This is unlikely. Following interviews, in an effort to ensure data accuracy and to seek additional information, region results were mailed to Chiefs of Surgery at all hospitals in the respective region, and to other stakeholders such as Cancer Care Ontario Regional Vice Presidents. Very little additional data were forthcoming, and no additional region-level interventions or relevant implementation processes were identified. Fourth, there may have been a bias to over-report KT activities by some interviewees (e.g., regional surgical oncology and colorectal surgery leads) since the mandate of such individuals was to implement activities encouraged by Cancer Care Ontario. However, KT signature scores in the second group of 12 regions were quite low with an average score of 30.5 (range 22–38). Such low scores do not suggest over-reporting of KT activities. The lowest score possible was 20 reflecting no region-level activity. Fifth, we did not evaluate the fidelity of KT intervention delivery. This is currently a topic of great interest among KT researchers though there are no reports of evaluating implementation fidelity at a region or population level [21]. It may be of interest to integrate intervention implementation fidelity into the next iteration of our KT Signature Assessment Tool. Finally, scores for implementation of KT interventions at a region-level may not reflect impact on patient care, or the effectiveness of said interventions. As mentioned, we plan to compare process and outcome measures for KT Signature I versus II regions. This should provide insights on whether our KT intervention implementation scores correlate with improved quality of care.

## Conclusion

We used interviews with stakeholders across Ontario, a novel KT Signature Assessment Tool, and a Delphi process with KT experts, to audit and score the implementation of KT interventions used to improve the quality of rectal cancer surgery in each of 14 Ontario health regions. The distribution of scores was bi-modal. Two regions had numerous interventions that were iterative, informed by data, and initiated by front-line surgeons while the other 12 regions executed – often in a limited fashion - interventions that originated from Cancer Care Ontario in a top-down manner. These two groupings will be used for future quantitative analyses to help determine if higher scores are commensurate with superior patient process and outcome measures. These methods and findings require validation, though they should be of interest to researchers and stakeholders attempting to audit and score KT interventions implemented at a region level for other aspects of clinical care.

## Supplementary information

**Additional file 1.** Interview Guide.

## Data Availability

The datasets used and/or analysed during the current study are available from the corresponding author on reasonable request.

## Abbreviations

KT: knowledge translation
iKT: integrated knowledge translation
KTA cycle: knowledge to action cycle
