# Supplementary material for: A method to audit and score implementation of knowledge translation (KT) interventions in large health regions – an observational pilot study using rectal cancer surgery in Ontario
Source: BMC Health Serv Res. 2020 Jun 5;20:506. doi: 10.1186/s12913-020-05353-9 (PMC7275399; doi:10.1186/s12913-020-05353-9)
Supplement: Supplementary file 1 — Additional file 1. Interview Guide. [file 12913_2020_5353_MOESM1_ESM.doc]

**Introduction:**

Thank you for agreeing to participate in an interview to discuss quality improvement activities related to colorectal cancer surgery.

We are conducting interviews with surgeons and administrators across the province with the aim to create a comprehensive description of the QI activities that have been initiated in LHINs over the past 5 to 10 years.

The interview will focus on any QI activities related to colorectal cancer surgery that have been started in your LHIN.

To facilitate the interview, we included with your letter a list of recognized QI strategies.

I will refer to this list as we discuss if any quality improvement activities have been going on in your LHIN.

Do you have the list COLOURED PAPER handy?

Let’s get started.

Perhaps we can start by talking about any activities in your LHIN specifically directed at **identifying quality gaps**.

*[Identify the quality gap(s)?]*

1. Did your LHIN identify quality gaps or areas related to colorectal cancer surgery that you wanted to improve or address?

**PROBE TO IDENTIFY SPECIFIC GAP(S)**: CRM margins, use of pre-/post-op radiation, use of CT/MRI, waiting times for surgery

1. How did your LHIN identify the quality gap or select this issue as an area to focus on?

**PROBE TO IDENTIFY HOW THEY DID THIS)**: Did you use clinical data specific to your LHIN, clinicians’ perceptions, provincial clinical data?

1. Who was involved in identifying is area?
2. As part of your work, did you assess barriers to quality? In other words, did you carry out any activities like interviews, focus groups, or surveys to identify factors that contribute to this quality gap?

**EXAMPLES OF BARRIERS:** Lack of awareness or knowledge around the issue; habitual practices (i.e., we’ve always done it this way); organizational barriers (e.g. resource issues).

Next, let’s turn to the **handout listing the QI activities**.

The first activity area that we will discuss is **Communities of Practice**….

**QUESTIONS - COMMUNITIES OF PRACTICE**

A CoP is a group of people who share knowledge, learn together, and create common practices.[[1]](#footnote-2)

Your LHIN may have established a CoP based on the Cancer Care Ontario initiative described below, or, your CoP may have other activities that you tailored for your region.

**PREAMBLE**

Surgical Oncology Program at CCO

In 2010, a province-wide colorectal CoP was established by CCO to identifying gaps in cancer care and develop quality improvement activities to address these gaps. The colorectal CoP is composed of leaders from each region who share current practices and challenges to move quality initiatives forward. Members are encouraged to develop CoPs within their own regions that encompasses multiple physician disciplines and hospitals from within the region.[[2]](#footnote-3)

CCO encourages CoPs, and there have been numerous strategies implemented at a provincial level to engage members of the colorectal cancer CoPs. These include:

***List Serv Online Discussion Forum***: The Colorectal and Prostate Cancer List Servs provided a multidisciplinary online discussion platform for physicians/COP members to improve their knowledge and management of colorectal cancer.

***Provincial Workshops***: In-person workshops and web-conferences provide an opportunity for multiple disciplines from across the province to come together and discuss quality improvement initiatives, identify gaps in care, and determine goals for the COP.

***Newsletters***: Newsletters keep COP members informed of quality improvement initiatives occurring in the province.

***Data Distribution***: Data is an important driver of quality improvement, and both the colorectal cancer and prostate cancer COPs receive data on *positive margin rates* and *lymph node status*.

***Educational Slide decks***: Presentations on the colorectal and prostate cancer guidelines can be used at regional educational rounds or meetings.

**QUESTIONS**

Has your LHIN formally established a COMMUNITY OF PRACTICE at the regional or LHIN level?

If so, what activities comprise your CoP?

1. What role did the CCO initiative play in establishing your CoP?
2. Who took the leadership role, locally, to establish the CoP?
3. How is the CoP supported? Funded? What organization coordinates the CoP activities?

I think the best way for us to fully address the activities in your CoP is to discuss each one individually. Let’s start with _______________________________.

Our next intervention is **EDUCATIONAL MEETINGS**

This includes things like conferences, lectures, or workshops.

**Examples**

Morbidity & Mortality (M & M Rounds) Regional or hospital workshops

Journal club Other rounds (e.g., research)

**QUESTIONS – CANCER CARE ONTARIO SUPPORTED WORKSHOPS**

1. Are you aware that funding is available through Cancer Care Ontario for quality workshops or meetings at the LHIN level?
2. Have you hosted any workshops in your LHIN specifically for colorectal cancer surgery that were supported by CCO?
3. Was it a LHIN-wide workshop or at the hospital level?
4. How many have you had in the past 5 years or so?
   - Dates and locations

***For each of the workshops, we are interested in knowing the topic areas discussed. Do you have meeting packages and minutes that you would be willing to provide to the study team?***

**Select, tailor, implement**

1. What quality area(s) did the workshop address?
   - What was the title or focus of the workshop?
2. Why did you select a workshop as the intervention to address this quality gap?
   - Did you base the decision to use a workshop on evidence or theory or practicality?
3. Who hosted the workshop?
4. What was your target audience for workshop?
5. Was it strictly general surgeons? Were other clinicians (pathologists, radiologists) or administrators invited?
6. What was the participation rate in the workshop?
7. Tell me about the process used to select the focus for the workshop.
   - Who took the lead on organizing the workshop?
   - What role did the target audience play in planning the workshop agenda?

**Monitor knowledge use**

1. Did any recommendations come out of the workshop?
   - Are these documented in the minutes?
2. Do you measure progress related to these recommendations?
3. If so, how do you do that?
   - Do you collect data?
   - Who is responsible for interpreting and presenting the data?
   - How is the data shared with your target audience?)
4. What role does the target audience play in the monitoring process?

**Evaluate outcomes**

1. Have you achieved improvement related to the areas identified in the workshop recommendations?
   - In what areas – processes of care, patient outcomes?

**QUESTIONS – M & M ROUNDS**

1. Do you have M & M rounds in your LHIN? Are these at the hospital-level? Or LHIN-level?

**Select, tailor, implement**

1. How often are these rounds (e.g., monthly, quarterly) Or, as needed?
2. Who attends the rounds?
   - Is attendance mandatory?
   - What are your participation rates in these rounds?
3. Who decides on the cases for presentation?
   - What role do the surgeons, or other clinicians play in planning the rounds?
4. Do specific recommendations specific to quality issues come out of these rounds?
   - How are the recommendations communicated to the target group [surgeons]?
5. Do you have meeting packages and minutes that you would be willing to provide to the study team?

**Monitor knowledge use**

1. Do you measure progress related to these recommendations or quality issues?

If so, how do you do that?

- - Do you collect data?
  - Who is responsible for interpreting and presenting the data?
  - How is the data shared with your target audience?

1. What role does the target audience play in the monitoring process?

**Evaluate outcomes**

1. Have you achieved improvement related to the areas identified in the areas identified in your recommendations?

In what areas – processes of care, patient outcomes?

1. Do you have any reports, publications, presentations that you can share with our research team regarding these successes?

**QUESTIONS – OTHER EDUCATIONAL MEETINGS**

1. Do you, or have you hosted any other educational lectures, or meetings, or conferences about colorectal cancer surgery?
2. If so, what kind of meeting was it?
3. Was this a LHIN-wide meeting or for a specific hospital?

**Select, tailor, implement**

1. What quality area(s) did the meeting address?
   - What was the title or focus of the meeting?
2. Who hosted the meeting?
3. What was your target audience for meeting?
   - Was it strictly general surgeons? Were other clinicians (pathologists, radiologists) or administrators invited?
4. What was the participation rate in the meeting?
5. Tell me about the process used to select the focus for the meeting.
   - Who took the lead on organizing the meeting?
   - What role did the target audience play in planning the meeting agenda?
6. What were the learning objectives for the meeting?
7. Why did you select a meeting as the intervention to address this quality gap?
   - Did you base the decision to use a workshop on evidence or theory or practicality?
8. Do you have meeting packages and minutes that you would be willing to provide to the study team?

**Monitor knowledge use**

1. Did any recommendations come out of the meeting? [
   - Are these documented in the minutes?
2. Do you measure progress related to these recommendations?
3. If so, how do you do that?
   - Do you collect data?
   - Who is responsible for interpreting and presenting the data?
   - How is the data shared with your target audience?
4. What role does the target audience play in the monitoring process?

**Evaluate outcomes**

1. Have you achieved improvement related to the areas identified in the meeting recommendations?
   - In what areas – processes of care, patient outcomes?
   - Do you have any reports, publications, presentations that you can share with our research team regarding these successes?

*********************************************************************************************************************************

**CHECK TO ENSURE ALL OF THESE AREAS WERE ADDRESSED**

**Have you implemented any of the specific activities outlined below to address the quality gaps you identified?**

- - Why did you select it? What quality gap does it address?
  - Is (was) the activity a LHIN-wide initiative or specific segment or portion of the LHIN or just for a particular hospital(s)? Is (was) this activity organized by leaders of the LHIN (versus a specific hospital)?
  - Is (was) this activity a sponsored/endorsed activity?
  - Who is the target audience for the activity? How many individuals of your target audience were involved? What role did the target group play in selecting the activity?
  - How often does (did) the activity occur? (e.g., ongoing, at regular intervals, one time only)

**How do you measure changes related to implementation of the activity? (E.g., participation rates in workshops, measure the use of preoperative CT or MRI, monitor changes in waiting lists)**

- - What sort of measurement activities did you do?
  - Do you get feedback from participants about the activity in terms of its quality, its effectiveness
  - How did you measure this? Are you planning to measure this in the future?

**Has this strategy made a difference on desired outcomes for patients, practitioners, or the system?**

**DISTRIBUTION OF EDUCATIONAL MATERIALS**

**Definition:**

This includes “*the distribution of published or printed recommendations for clinical care, including clinical practice guidelines, audio-visual materials and electronic publications. The materials may have been delivered personally or through mass mailings.”*

**CCO produced guidelines** on cross-sectional imaging (2006), multidisciplinary case conferences (2006), and, surgical and pathology standards related to margins and lymph nodes (2008).

**References:**

Simunovic M, Stewart L, Zwaal C, et al. **Cross-sectional imaging** in colorectal cancer: recommendations report. Toronto, Canada: Cancer Care Ontario (CCO); 2006. Website: http://www.cancercare.on.ca/pdf/pebcdicrc.pdf (accessed 2011 Sept. 6).

F. Wright, C. De Vito, B. Langer, A. Hunter. **Multidisciplinary Cancer Conference Standards**: special report. Toronto, Canada: Cancer Care Ontario (CCO); 2006. Website: http://www.cancercare.on.ca/common/pages/UserFile.aspx?fileId=14320 (accessed 2011 Sept. 6).

Smith AJ, Driman DK, Spithoff K, et al.: Optimization of surgical and pathological quality performance in radical surgery for colon and rectal cancer: **Margins and lymph nodes**; 2008 [cited 2009 August 10]. Website: http://www.cancercare.on.ca/common/pages/UserFile.aspx?fileId=13954(accessed 2011 Sept. 6).

A. Smith, R.B. Rumble, B. Langer, H. Stern, F. Schwartz, M. Brouwers, and members of Cancer Care Ontario’s Laparoscopic Colon Cancer Surgery Expert Panel and Program in Evidence-based Care. **Laparoscopic Surgery for Cancer of the Colon**. Report Date: September 2005 This Evidence-based Series (EBS) was reviewed in September 2011 and ARCHIVED in 2012.

**QUESTIONS**

Are you familiar with these CCO guideline(s)/article(s)?

Was there any formal process to distribute these guideline(s)/article(s) to surgeons or other groups in your region?

**If YES, proceed with the question page.**

Publication specific to quality indicators for colorectal cancer surgery

Gagliardi AR, Simunovic M, Langer B, Stern H, Brown AD. [Development of quality indicators for colorectal cancer surgery, using a 3-step modified Delphi approach.](http://www.ncbi.nlm.nih.gov.libaccess.lib.mcmaster.ca/pubmed/16417050) Can J Surg. 2005 Dec;48(6): 441-52.

**QUESTIONS**

Are you familiar with this article?

Was there any formal process to distribute the article to surgeons or other groups in your region?

**If YES, proceed with the question page.**

Other articles or reports distributed in your LHIN.

**QUESTIONS**

Have you distributed any other educational materials or articles in your region?

**If YES, proceed with the question page.**

**QUESTIONS**

1. Are you familiar with these CCO guideline(s)/article(s)?
2. Was there any formal process to distribute these guideline(s)/article(s) to surgeons or other groups in your region?

**If YES, proceed with the questions below, if NO skip to “OTHER EDUCATIONAL MATERIALS”**

**Identify the quality gap(s)?**

1. Why did your LHIN decide to distribute the guideline(s)/article(s)? What prompted your region to distribute the guideline(s)/article(s)?

**Adapt knowledge to local user**

1. Did you distribute the guideline(s)/article(s)as published, or did you adapt it in any way to better fit your local context?
2. If so, in what way? What was the rationale behind the adaptation?
3. Did the target audience participate in this process?

**Select, tailor, implement intervention**

1. To whom did you distribute the guideline(s)/article(s)? (surgeons, radiologists, pathologists, others)
2. How did you distribute them?
3. Did you distribute the guideline(s)/article(s)across the LHIN (or region)? Or, just to particular hospitals, or selected colleagues.
4. When did you distribute the guideline(s)/article(s)? When did this occur relative to the release of each guideline(s)/article(s)?
5. How was the guideline(s) distributed? [By mail, email, via website, as part of a workshop package]
6. Who distributed the guideline(s)? What person or organization was responsible for distributing the material(s)?

**Monitor knowledge use**

1. Was there a formal process or forum organized to review the guideline(s)/article(s)? Who attended the forum? What was the purpose of the forum – was it to review the content? Was it to develop a strategy to implement the guidelines?
2. Are there minutes or notes from the forum? Would it be possible to obtain a copy of these minutes?
3. Were people interested in the guideline(s)/article(s)? Was it/were they well received by the group?
4. Did the group identify any barriers or issues that would hinder implementation of the guideline? If so, what did they identify? How did you address their concerns?

**Evaluate outcomes**

1. Has distributing the guideline(s)/article(s) made a difference on desired outcomes for patients, practitioners, or the system?
2. Did your group set targets for improvement related to the guideline(s)/article(s)?
3. Do you measure this clinical practice (e.g. use of preoperative CTs or MRIs) or patient outcome (e.g. local recurrence)? If YES:
   - What data does your group collect?
   - How do you collect this data?
   - Who is responsible for interpreting and presenting these data?
   - Who gets the data reports?
4. Have you achieved success related to distribution of the guideline(s)/article(s)? (E.g., improved clinical practice like decreased rates of positive radial margins)
5. For how long have you monitored processes of care or patient outcomes?
6. Do you have any reports, publications, presentations that you can share with our research team regarding these successes?

**Sustain knowledge use**

1. Has these guideline(s)/article(s) been redistributed at any point?
2. When was it redistributed? For what purpose?

*********************************************************************************************************************************

**CHECK TO ENSURE ALL OF THESE AREAS WERE ADDRESSED**

**Have you implemented any of the specific activities outlined below to address the quality gaps you identified?**

- - Why did you select it? What quality gap does it address?
  - Is (was) the activity a LHIN-wide initiative or specific segment or portion of the LHIN or just for a particular hospital(s)? Is (was) this activity organized by leaders of the LHIN (versus a specific hospital)?
  - Is (was) this activity a sponsored/endorsed activity?
  - Who is the target audience for the activity? How many individuals of your target audience were involved? What role did the target group play in selecting the activity?
  - How often does (did) the activity occur? (e.g., ongoing, at regular intervals, one time only)

**How do you measure changes related to implementation of the activity? (E.g., participation rates in workshops, measure the use of preoperative CT or MRI, monitor changes in waiting lists)**

- - What sort of measurement activities did you do?
  - Do you get feedback from participants about the activity in terms of its quality, its effectiveness
  - How did you measure this? Are you planning to measure this in the future?

**Has this strategy made a difference on desired outcomes for patients, practitioners, or the system?**

Let’s move on to the next intervention, **AUDIT AND FEEDBACK**

Audit and feedback involves measuring clinical performance or patient outcomes (e.g., your clinical practice, that of your team or that of your LHIN) together with comparative data (e.g., data from other clinicians or other LHINs, etc.) and reporting the data back to participants. The information might be collected from medical records or computerized databases.

**Examples**

Cancer Care Ontario publishes various quality indicators at the LHIN-level as part of the **Cancer System Quality Index** (CSQI) 2011. [Website: http://www.csqi.on.ca/cms/one.aspx accessed September 13, 2011].

Indicators for prevention, screening, diagnosis, treatment, recovery and end of life - Broken down by LHIN

And, wait times for cancer surgery

Relevant indicators on the CSQI for colorectal cancer:

Percentage of colon cancer resection reports with 12 or more lymph nodes examined

Percentage of Stage III colon cancer patients who consult with a medical oncologist within 4 months of

Percentage of rectal cancer surgery reports indicating a positive circumferential margin

Percentage of Stage III colon cancer patients aged 65 or over treated with guideline recommended chemotherapy

Percentage of cancer patients having surgery who are treated within recommended target wait times

Adherence to standards criteria of reported Multidisciplinary Case Conferences

Wait Times - Percentage of cases completed within priority access

**QUESTIONS**

- - 1. Are you familiar with the **Cancer System Quality Index** published on the CCO website?

**If YES, proceed with the question page.**

- - 1. **Surgical Quality Reports** prepared by Cancer Care Ontario are disseminated quarterly with surgeon leads. The reports present LHIN-based performance data together with provincial quality targets.

**QUESTIONS**

Are you familiar with the distribution of these reports via the surgical lead in your LHIN?

**If YES, proceed with the question page.**

- - 1. **Your LHIN may collect data its own data on selected performance or outcomes measures that is shared with surgeons.**

**QUESTIONS**

Do you, or have you collected data related to colorectal cancer surgery that you share with surgeons or other clinicians in your LHIN?

**If YES, proceed with the question page.**

**QUESTIONS - CANCER SYSTEM QUALITY INDEX PUBLISHED ON THE CCO WEBSITE**

**Select, tailor, implement**

1. Have you been on the website?
2. Was there, or is there currently, a formal process to make surgeons in your region aware of the CCO quality indicators website?
3. What prompted your region to alert your surgeons to this website?
4. Who took the lead on this?

**Adapt knowledge to local context**

1. Do you distribute data from this website to surgeons in your region? If so, what prompted your region to distribute this data?
   - How are the data distributed? [By mail, email, via website, as part of a workshop package]
   - Is it possible to get a copy of what was distributed?
   - To whom do you distribute the indicators/wait times report? (surgeons, hospital administrators, other clinical groups such as pathologists, radiologists, nurses)
   - Is the material distributed across the LHIN? Or, just to particular hospitals?
   - Who distributes the indicators/wait times reports?
   - Is there any financial or in-kind support to distribute the material(s)?

**Select, tailor, implement**

1. Is there a formal process or meeting organized to review the CCO indicators or wait times?
2. Who is invited or attends the meeting?
3. Are there minutes or notes from the meeting(s)? Would it be possible to obtain a copy of these minutes?

**Monitor Knowledge Use**

1. Are surgeons interested in the CCO indicators?

**Evaluate Outcomes**

1. Did your group set targets for improvement related to the CCO indicators or wait times?
2. Do you measure any of these CCO indicators locally (e.g. lymph node counts or CRM rates) If YES:
   - What data does your group collect?
   - How do you collect this data?
   - Who is responsible for interpreting and presenting these data?
   - Who gets the data reports?
3. Do you have any reports, publications, presentations that you can share with our research team regarding these successes?

*********************************************************************************************************************************

**CHECK TO ENSURE ALL OF THESE AREAS WERE ADDRESSED**

**Have you implemented any of the specific activities outlined below to address the quality gaps you identified?**

- - Why did you select it? What quality gap does it address?
  - Is (was) the activity a LHIN-wide initiative or specific segment or portion of the LHIN or just for a particular hospital(s)? Is (was) this activity organized by leaders of the LHIN (versus a specific hospital)?
  - Is (was) this activity a sponsored/endorsed activity?
  - Who is the target audience for the activity? How many individuals of your target audience were involved? What role did the target group play in selecting the activity?
  - How often does (did) the activity occur? (e.g., ongoing, at regular intervals, one time only)

**How do you measure changes related to implementation of the activity? (E.g., participation rates in workshops, measure the use of preoperative CT or MRI, monitor changes in waiting lists)**

- - What sort of measurement activities did you do?
  - Do you get feedback from participants about the activity in terms of its quality, its effectiveness
  - How did you measure this? Are you planning to measure this in the future?

**Has this strategy made a difference on desired outcomes for patients, practitioners, or the system?**

**QUESTIONS - SURGICAL QUALITY REPORTS PREPARED BY CANCER CARE ONTARIO ARE DISSEMINATED QUARTERLY WITH SURGEON LEADS**

**Select, tailor, implement**

1. Are you familiar with these quarterly reports?
2. Is there any formal process to make surgeons in your region aware of the reports?

**Adapt knowledge to local context**

1. Are these reports distributed to surgeons in your region? If so:
   - When did you start to distribute these reports?
   - How are the reports distributed? [By mail, email, via website, as part of a workshop package]
   - To whom do you distribute the reports? (surgeons, hospital administrators, other clinical groups such as pathologists, radiologists, nurses)
   - Is the material distributed across the LHIN? Or, just to particular hospitals?
   - Who distributes the reports?

**Select, tailor, implement**

1. Is there a formal process or meeting organized to review the reports?
2. Who is invited or attends the meeting?

**Monitor Knowledge Use**

1. Are surgeons interested in these reports?

**Evaluate Outcomes**

1. Did your group set targets for improvement related to the information in the reports?

*********************************************************************************************************************************

**CHECK TO ENSURE ALL OF THESE AREAS WERE ADDRESSED**

**Have you implemented any of the specific activities outlined below to address the quality gaps you identified?**

- - Why did you select it? What quality gap does it address?
  - Is (was) the activity a LHIN-wide initiative or specific segment or portion of the LHIN or just for a particular hospital(s)? Is (was) this activity organized by leaders of the LHIN (versus a specific hospital)?
  - Is (was) this activity a sponsored/endorsed activity?
  - Who is the target audience for the activity? How many individuals of your target audience were involved? What role did the target group play in selecting the activity?
  - How often does (did) the activity occur? (e.g., ongoing, at regular intervals, one time only)

**How do you measure changes related to implementation of the activity? (E.g., participation rates in workshops, measure the use of preoperative CT or MRI, monitor changes in waiting lists)**

- - What sort of measurement activities did you do?
  - Do you get feedback from participants about the activity in terms of its quality, its effectiveness
  - How did you measure this? Are you planning to measure this in the future?

**Has this strategy made a difference on desired outcomes for patients, practitioners, or the system?**

**QUESTIONS – AUDIT & FEEDBACK INITIATED WITHIN THE LHIN**

**Select, tailor, implement**

1. Why did you select audit and feedback as an activity?
2. What quality issue(s) does this activity address?
3. What data do you collect?
   - Do you measure clinical processes or practice (e.g. use of preoperative CTs or MRIs)?
   - Do you measure patient outcomes (e.g., in-hospital mortality, local recurrence)?
4. At what level do you collect this data? (hospital-level, regional or LHIN-level, care-provider-level, etc)
5. When did the intervention start?
   - When was your first audit?
   - Is the audit ongoing, or point in time or snapshot?
6. Who actually collects the data?
   - What individual/group/ organization oversees the audit and feedback process?
7. What are the data sources?
8. What quality area does this audit and feedback address?
9. Who is the target audience for the data? (general surgeons, pathologists, radiologists, policy makers)?
10. In what format do you feedback this information? (written report, website, presentation)
    - To whom do you report the data?
    - Do you include comparator data? (E.g., other clinicians, other hospitals, other regions)
    - Do you include trend data? (E.g., previous time periods)
    - **Is the data fed back to surgeons as a group or do you meet one-on-one to review individual data? NOTE THAT INDIVIDUAL MEETINGS SHOULD BE PURSUED AS ACADEMIC DETAILING**
11. At what intervals do you feedback this data?
    - Is it ongoing? Or, at regular intervals (e.g., monthly, quarterly) Or, as needed?
12. Is any financial support or in-kind support provided for this intervention?
13. Did you learn about auditing this data from another hospital or region?
    - Or, did the idea to collect this data originate in your LHIN?
    - If the idea originated in another hospital or organization, did you change it in any way to better suit your target audience? If so, what adjustments did you make?

Monitor knowledge use

1. Are people interested in the data?
2. Has the group identified other interventions to address quality gaps identified by the data?
   - If so, what did they identify? Have these been implemented? *(note these interventions and discuss under relevant topic area)*

Evaluate outcomes

1. Do you have established targets for improvement (benchmarks) for your audit and feedback data?
   - How were these targets established?
   - What role did the surgeons or target audience play in establishing the targets?
2. Have you achieved improvement in the areas that you monitor with audit and feedback?
   - In what areas – processes of care, patient outcomes?
   - Do you have any reports, publications, presentations that you can share with our research team regarding these successes?

*********************************************************************************************************************************

**CHECK TO ENSURE ALL OF THESE AREAS WERE ADDRESSED**

**Have you implemented any of the specific activities outlined below to address the quality gaps you identified?**

- - Why did you select it? What quality gap does it address?
  - Is (was) the activity a LHIN-wide initiative or specific segment or portion of the LHIN or just for a particular hospital(s)? Is (was) this activity organized by leaders of the LHIN (versus a specific hospital)?
  - Is (was) this activity a sponsored/endorsed activity?
  - Who is the target audience for the activity? How many individuals of your target audience were involved? What role did the target group play in selecting the activity?
  - How often does (did) the activity occur? (e.g., ongoing, at regular intervals, one time only)

**How do you measure changes related to implementation of the activity? (E.g., participation rates in workshops, measure the use of preoperative CT or MRI, monitor changes in waiting lists)**

- - What sort of measurement activities did you do?
  - Do you get feedback from participants about the activity in terms of its quality, its effectiveness
  - How did you measure this? Are you planning to measure this in the future?

**Has this strategy made a difference on desired outcomes for patients, practitioners, or the system?**

**QUESTIONS - PRACTICE DEMONSTRATIONS**

**Definition:**

A practice demonstration is when an expert goes to the surgeon’s, or clinician’s hospital to demonstrate a skill or technique.

**QUESTIONS - CANCER CARE ONTARIO & the Ontario Association of General Surgeons (OAGS)**

An example of a practice demonstration would be the Laparoscopic Colon Mentoring Program that was initiated by CCO and then transferred to the OAGS. (2007-08)

**Select, tailor, implement**

1. Did surgeons in your LHIN participate in this mentoring program?
   - What prompted your LHIN to participate in this program?

**Monitoring Knowledge Use**

1. Approximately how many surgeons participating in the CCO/OAGS mentoring?
2. Did your group set any targets for improvement as a result of participating in the mentoring?

**Evaluate Outcomes**

1. Did you measure progress related to the mentoring?
   - Do you measure clinical processes or practice (e.g. use of preoperative CTs or MRIs)?
   - Do you measure patient outcomes (e.g., in-hospital mortality, local recurrence)?
   - What data do you collect?
   - How do you collect your data?
   - Who is responsible for interpreting and presenting the data?
   - How is the data shared with your target audience?
2. Have you achieved improvement related to the laparoscopic surgery mentoring?
   - In what areas – processes of care, patient outcomes?
   - Do you have any reports, publications, presentations that you can share with our research team regarding these successes?

**Efforts to sustain knowledge use**

1. Have you offered any continuation of the laparoscopic mentoring in your area? Or have you offered any related education or interventions about laparoscopic colon surgery?

*********************************************************************************************************************************

**CHECK TO ENSURE ALL OF THESE AREAS WERE ADDRESSED**

**Have you implemented any of the specific activities outlined below to address the quality gaps you identified?**

- - Why did you select it? What quality gap does it address?
  - Is (was) the activity a LHIN-wide initiative or specific segment or portion of the LHIN or just for a particular hospital(s)? Is (was) this activity organized by leaders of the LHIN (versus a specific hospital)?
  - Is (was) this activity a sponsored/endorsed activity?
  - Who is the target audience for the activity? How many individuals of your target audience were involved? What role did the target group play in selecting the activity?
  - How often does (did) the activity occur? (e.g., ongoing, at regular intervals, one time only)

**How do you measure changes related to implementation of the activity? (E.g., participation rates in workshops, measure the use of preoperative CT or MRI, monitor changes in waiting lists)**

- - What sort of measurement activities did you do?
  - Do you get feedback from participants about the activity in terms of its quality, its effectiveness
  - How did you measure this? Are you planning to measure this in the future?

**Has this strategy made a difference on desired outcomes for patients, practitioners, or the system?**

**QUESTIONS - EDUCATIONAL OUTREACH or ACADEMIC DETAILING**

**Definition:**

Educational outreach or academic detailing as “use of a trained person who meets with providers in their practice settings to give information with the intent of changing the providers’ practice. The information given may include feedback on the performance of the provider(s).”

[Educational outreach is when a trained person meets with providers to review practice, or give feedback on performance.]

**NOTE THAT THIS AREA MAY HAVE BEEN IDENTIFIED AS AN INTERVENTION AS PART OF AUDIT AND FEEDBACK**

**QUESTIONS**

**Context of Implementation**

As I understand it, you meet with individual surgeons to go over data/information related to ______

1. What quality gap does intervention address?
   - How did your group become aware of this quality gap?
2. Is this done across your LHIN, or just with particular surgeons or hospitals?

**Select, tailor, implement**

1. Tell me about the process used to select academic detailing (going out to present data or feedback to individual surgeons).
   - Who took the lead on selecting this intervention?
2. Why you opt to meet on-on-one with surgeons?
   - What was the evidence to support using this intervention?
3. What role did the surgeons play in the selecting the intervention?
4. Did you develop this intervention or did you learn from another hospital or organization?
   - If your intervention originated in another hospital or organization did you change it in any way to better suit your target audience?
   - If so, what adjustments did you make?
5. Is there any financial in-kind support provided for academic detailing?
6. Who coordinates the visits? (for example, helps to set up visits)
7. Who goes out to meet with the surgeons?
8. When did the intervention start? Are you still doing it?

**Monitor Knowledge Use**

1. Are surgeons receptive to these one-on-one meetings?
2. Do you contact surgeons to participate, or is it the surgeon who initiates the request?
3. What are your overall targets for improvement associated with these visits?

**Evaluate outcomes**

1. How do you measure improvements associated with this outreach intervention?
   - Do you measure clinical processes or practice (e.g. use of preoperative CTs or MRIs)? Do you measure patient outcomes (e.g., in-hospital mortality, local recurrence)?
   - What data do you collect? How is the data shared with the surgeons that you meet with?
2. Have you achieved improvement as a result of this outreach?
   - In what areas – processes of care, patient outcomes?
3. Do you have any reports, publications, presentations that you can share with our research team regarding these successes?

*********************************************************************************************************************************

**CHECK TO ENSURE ALL OF THESE AREAS WERE ADDRESSED**

**Have you implemented any of the specific activities outlined below to address the quality gaps you identified?**

- - Why did you select it? What quality gap does it address?
  - Is (was) the activity a LHIN-wide initiative or specific segment or portion of the LHIN or just for a particular hospital(s)? Is (was) this activity organized by leaders of the LHIN (versus a specific hospital)?
  - Is (was) this activity a sponsored/endorsed activity?
  - Who is the target audience for the activity? How many individuals of your target audience were involved? What role did the target group play in selecting the activity?
  - How often does (did) the activity occur? (e.g., ongoing, at regular intervals, one time only)

**How do you measure changes related to implementation of the activity? (E.g., participation rates in workshops, measure the use of preoperative CT or MRI, monitor changes in waiting lists)**

- - What sort of measurement activities did you do?
  - Do you get feedback from participants about the activity in terms of its quality, its effectiveness
  - How did you measure this? Are you planning to measure this in the future?

**Has this strategy made a difference on desired outcomes for patients, practitioners, or the system?**

**QUESTIONS – PRACTICE DEMONSTRATIONS INITIATED WITHIN THE LHIN**

**Select, tailor, implement**

1. Have you initiated any similar types of practice demonstrations for colorectal cancer surgery in your LHIN?
2. Describe your practice demonstration intervention.
3. Is this a LHIN-wide intervention, or at one hospital only?
   - Who does the demonstration target?
4. Who provides the demonstrations (trained persons, or experts)?
   - How were they chosen?
   - What are their credentials with regard to this intervention? (E.g., specialized surgical training)
5. What quality gap does intervention address? How did your group become aware of this quality gap?
6. Why did you select practice demonstrations to address this knowledge gap?
   - Did you pick demonstrations based on evidence (positive impact of demonstrations)? Or practicality?
7. Did you develop this intervention or did you learn from another hospital, or organization?
   - If your intervention originated in another hospital or organization did you change it in any way to better suit your surgeons?
   - If so, what adjustments did you make?
8. Who coordinates the demonstrations? (for example, helps to set up visits)
   - Do you contact the surgeons? Or, is it up to surgeons to request a demonstration? Or both?
9. When did the intervention start?
   - When was the first demonstration? Is it still ongoing?
10. Is any financial or in-kind support provided for outreach?

**Monitoring Knowledge Use**

1. How many demonstrations have been done?
   - How many surgeons have participated?
2. What are your overall aims and targets for improvement as a result of these demonstrations?

**Evaluate Outcomes**

1. How do you measure progress related to these demonstrations?
   - Do you measure clinical processes or practice (e.g. use of preoperative CTs or MRIs)?
   - Do you measure patient outcomes (e.g., in-hospital mortality, local recurrence)?
   - What data do you collect?
   - How do you collect your data?
   - Who is responsible for interpreting and presenting the data?
   - How is the data shared with your target audience?
2. Have you achieved improvement related to these demonstrations?
   - In what areas – processes of care, patient outcomes?
   - Do you have any reports, publications, presentations that you can share with our research team regarding these successes?

**Efforts to sustain knowledge use**

1. Do you do follow up visits or demonstrations with participants?
2. Is this an ongoing activity? Still going on in the LHIN?

*********************************************************************************************************************************

**CHECK TO ENSURE ALL OF THESE AREAS WERE ADDRESSED**

**Have you implemented any of the specific activities outlined below to address the quality gaps you identified?**

- - Why did you select it? What quality gap does it address?
  - Is (was) the activity a LHIN-wide initiative or specific segment or portion of the LHIN or just for a particular hospital(s)? Is (was) this activity organized by leaders of the LHIN (versus a specific hospital)?
  - Is (was) this activity a sponsored/endorsed activity?
  - Who is the target audience for the activity? How many individuals of your target audience were involved? What role did the target group play in selecting the activity?
  - How often does (did) the activity occur? (e.g., ongoing, at regular intervals, one time only)

**How do you measure changes related to implementation of the activity? (E.g., participation rates in workshops, measure the use of preoperative CT or MRI, monitor changes in waiting lists)**

- - What sort of measurement activities did you do?
  - Do you get feedback from participants about the activity in terms of its quality, its effectiveness
  - How did you measure this? Are you planning to measure this in the future?

**Has this strategy made a difference on desired outcomes for patients, practitioners, or the system?**

**QUESTIONS - LOCAL OPINION LEADERS**

A local opinion leader is someone who is nominated by their colleagues as ‘educationally influential’ and whose role it is to share knowledge with regard to colorectal cancer surgery and influence their colleagues’ attitudes or clinical behavior in a positive way.

*Note that respondents may reference involvement in Andy Smith’s study on lymph nodes in which an opinion leader was selected as part of the study protocol. Or may note the QIRC Trial conducted by Simunovic et al (2002).*

**Select, tailor, implement**

1. Do you presently have, or have you ever had, a local opinion leader for colorectal cancer surgery?
2. When was an opinion leader first selected? Still in place? If not, why not?
3. What prompted your LHIN to select an opinion leader? Was implementation of an opinion leader based on theory or evidence?
4. Did you develop this intervention or did you learn from another hospital or organization or jurisdiction?
   - If you used a process from another hospital or jurisdiction, did you change the way you implemented the process [ to better suit your needs? If so, what adjustments did you make?
5. How was the opinion leader selected?
   - How were the opinion leader(s) selected? Who participated in the selection process? What role did the target audience play in the selecting the intervention?
6. Was an opinion leader chosen for each hospital? (or, for the LHIN)
7. What is the role of the opinion leader?
   - Is their role documented? (Obtain copy, if agreeable)
8. Is the opinion leader selected for a term or defined period of time?
9. What financial support is/was provided for this intervention? Any in-kind support?
10. What quality area does the opinion leader address?
    1. How did your group become aware of this quality gap?
11. Why was an opinion leader selected to address these areas?
    - Was the opinion leader intervention chosen for this area based on evidence? Or practicality?

**Monitoring knowledge use**

1. How does the opinion leader share information with the surgeons?
   - Formally? Informally?
   - Are these interactions documented?
2. What are the overall aims and targets for improvement?
3. Do you measure clinical processes or practice related to the purpose of [having] an opinion leader (e.g. use of preoperative CTs or MRIs)?
   - What data do you collect?
   - How do you collect your data?
   - Who is responsible for interpreting and presenting the data?
   - How is the data shared with your opinion leader and target audience?
4. Have you achieved improvement related to having an opinion leader?
   - In what areas – processes of care, patient outcomes?
   - Do you have any reports, publications, presentations that you can share with our research team regarding these successes?

*********************************************************************************************************************************

**CHECK TO ENSURE ALL OF THESE AREAS WERE ADDRESSED**

**Have you implemented any of the specific activities outlined below to address the quality gaps you identified?**

- - Why did you select it? What quality gap does it address?
  - Is (was) the activity a LHIN-wide initiative or specific segment or portion of the LHIN or just for a particular hospital(s)? Is (was) this activity organized by leaders of the LHIN (versus a specific hospital)?
  - Is (was) this activity a sponsored/endorsed activity?
  - Who is the target audience for the activity? How many individuals of your target audience were involved? What role did the target group play in selecting the activity?
  - How often does (did) the activity occur? (e.g., ongoing, at regular intervals, one time only)

**How do you measure changes related to implementation of the activity? (E.g., participation rates in workshops, measure the use of preoperative CT or MRI, monitor changes in waiting lists)**

- - What sort of measurement activities did you do?
  - Do you get feedback from participants about the activity in terms of its quality, its effectiveness
  - How did you measure this? Are you planning to measure this in the future?

**Has this strategy made a difference on desired outcomes for patients, practitioners, or the system?**

**QUESTIONS - REMINDERS**

Reminders are when clinically relevant information is provided verbally, on paper or is computer-generated, and is designed to prompt a health professional to remember to carry out important steps or procedures.

**QUESTIONS**

Have you implemented any reminder systems specifically related to colorectal cancer surgery?

**Select, tailor, implement**

1. Describe your reminder intervention.
2. What quality area does the reminder address?
   - How did your group become aware of this quality gap?
3. Tell me about the process used to select this reminder intervention.
   - Who took the lead on selecting this intervention?
   - Why did you select reminders as the intervention to address this quality area?
   - Was this based on evidence for reminders, or practicality?
4. In what format do you provide the reminder(s)? (computer, phone call/verbally, letter or email)
   - How is the reminder generated?
5. Who issues the reminder?
6. When did you first start sending out these reminders?
7. Who clinician group does the reminder target – surgeons? Pathologists? Nurses?
   - What role did the users play in planning and developing the reminder?
8. Is this a LHIN-wide system? Or just an individual hospital/department?
9. What role did the target audience – those who receive the reminder - play in the selecting this intervention?
10. Did you develop this intervention or did you learn from another hospital or jurisdiction?
    - If your intervention originated in another hospital or jurisdiction, did you change it in any way to better suit your users? If so, what adjustments did you make?
11. Do you have any financial support for this intervention? Any in-kind support?

**Monitor knowledge use**

1. Do you monitor follow through or responses to the reminders?
   - How do you monitor this?

**Evaluate Outcomes**

1. Have you experienced positive results with your reminder system?
   - Do you measure progress related to the reminder?
   - Do you measure specific clinical processes or practice (e.g. use of preoperative CTs or MRIs
   - What data do you collect?
   - How do you collect your data?
   - How is the data shared with your users?

*********************************************************************************************************************************

**CHECK TO ENSURE ALL OF THESE AREAS WERE ADDRESSED**

**Have you implemented any of the specific activities outlined below to address the quality gaps you identified?**

- - Why did you select it? What quality gap does it address?
  - Is (was) the activity a LHIN-wide initiative or specific segment or portion of the LHIN or just for a particular hospital(s)? Is (was) this activity organized by leaders of the LHIN (versus a specific hospital)?
  - Is (was) this activity a sponsored/endorsed activity?
  - Who is the target audience for the activity? How many individuals of your target audience were involved? What role did the target group play in selecting the activity?
  - How often does (did) the activity occur? (e.g., ongoing, at regular intervals, one time only)

**How do you measure changes related to implementation of the activity? (E.g., participation rates in workshops, measure the use of preoperative CT or MRI, monitor changes in waiting lists)**

- - What sort of measurement activities did you do?
  - Do you get feedback from participants about the activity in terms of its quality, its effectiveness
  - How did you measure this? Are you planning to measure this in the future?

**Has this strategy made a difference on desired outcomes for patients, practitioners, or the system?**

**QUESTIONS - TAILORED INTERVENTIONS**

**Tailored interventions** are intended to identify barriers to improvement or practice change – they use interviews or focus groups to identify challenges; and then, based on this information, strategies are developed to overcome the barriers and improve practice.

**Barriers to change** refer to factors that have the potential to impair the effectiveness of interventions designed to improve professional practice.

**QUESTIONS**

Have you carried out tailoring in your LHIN?

**Select, tailor, implement**

1. What prompted your hospital/LHIN/region to do tailoring?
2. Why did you select tailoring?
   - Did you select tailoring based on particular evidence or theory?
3. Did you develop this intervention or did you learn from another hospital or jurisdiction? If your intervention originated in another hospital, or jurisdiction, did you change it in any way to better suit your needs? If so, how?
4. When did you do tailoring?
   - Is tailoring ongoing? Or was it a time-limited process?
5. What approaches did you use?
   - Did you interview surgeons or other clinicians? Or a focus group?
   - If so, who was interviewed? (Surgeons? Pathologists? Nurses?)
   - How did you select the interviewees?
   - How many people participated in the interviews/part of the focus group?
6. Who conducted the interviews/focus group?
   - Why were they selected?
   - Who selected them?
7. Was tailoring done LHIN-wide? Or just an individual hospital/department?
8. What quality area did tailoring specifically address? Why did you choose to address this area?
   - Was there a particular practice area that you focused on?
9. Who analyzed and interpreted the data from the interviews/focus group?
10. Did specific recommendations come out of the analysis?
    - Who formulated the recommendations?
    - Did the target group participate in this process?
11. Were the results of the tailoring process formally communicated to your target audience? (report, at a meeting, workshops, website)

**Monitor knowledge use**

1. What are your targets for improvement related to this tailoring process?
2. What are your objectives for improvement?
3. Do you measure progress related to the tailoring process?
   - Do you measure clinical processes or practice (e.g. use of preoperative CTs or MRIs)?
   - Do you measure patient outcomes?
   - What data do you collect?
   - How do you collect your data?
   - Who is responsible for interpreting and presenting the data? How is the data shared with your target audience?
4. Have you achieved improvement in these areas?
   - In what areas – processes of care, patient outcomes?
   - Do you have any reports, publications, presentations that you can share with our research team regarding these successes?

*********************************************************************************************************************************

**CHECK TO ENSURE ALL OF THESE AREAS WERE ADDRESSED**

**Have you implemented any of the specific activities outlined below to address the quality gaps you identified?**

- - Why did you select it? What quality gap does it address?
  - Is (was) the activity a LHIN-wide initiative or specific segment or portion of the LHIN or just for a particular hospital(s)? Is (was) this activity organized by leaders of the LHIN (versus a specific hospital)?
  - Is (was) this activity a sponsored/endorsed activity?
  - Who is the target audience for the activity? How many individuals of your target audience were involved? What role did the target group play in selecting the activity?
  - How often does (did) the activity occur? (e.g., ongoing, at regular intervals, one time only)

**How do you measure changes related to implementation of the activity? (E.g., participation rates in workshops, measure the use of preoperative CT or MRI, monitor changes in waiting lists)**

- - What sort of measurement activities did you do?
  - Do you get feedback from participants about the activity in terms of its quality, its effectiveness
  - How did you measure this? Are you planning to measure this in the future?

**Has this strategy made a difference on desired outcomes for patients, practitioners, or the system?**

Our final area for discussion is **MULTIDISCIPLINARY CANCER CONFERENCE (MCC)**

**Definition:**

According to the CCO guideline published in 2006, “The intent of the multidisciplinary cancer conference (MCC) is to prospectively review individual cancer patients and make recommendations on best management, keeping in mind that individual physicians are responsible for making the ultimate treatment decision.

The guideline recommends that all cancer patients in Ontario should have the opportunity to have their case reviewed in an MCC.”

**Reference:**

F. Wright, C. De Vito, B. Langer, A. Hunter. **Multidisciplinary Cancer Conference Standards**: special report. Toronto, Canada: Cancer Care Ontario (CCO); 2006.

- Have you implemented a Multidisciplinary Case Conference for colorectal cancer in your LHIN?
- When did this start?
- Who initiated the MCC?

**MCC Cases**

- What cases are discussed at the MCC?
  - Are all cases newly diagnosed discussed at the MCC?
  - If not what cases are, or are not discussed?
  - Are there particular eligibility criteria?
- Who decides what cases are discussed?
- What role does the responsible individual physician have in determining if his/her case is discussed?
- Does the MCC have terms of reference, or a written protocol?
- Would it be possible for the study team to obtain a copy?

**Meeting Format**

- How often does the MCC meet?
- How long are the meetings?
- Approximately how many cases are reviewed at each conference?
- Does the MCC have a **designated Coordinator**?
  - What is his/her role in preparing for the meeting? During the meeting? Post-meeting?
- Who **chairs the MCC**? Is the Chair designated? Or, is it a rotating chair position?
  - What is the role of the Chair in preparing for the meeting? During the meeting? Post-meeting?
- Does the MCC have designated representatives from the following clinical areas?
  - Medical oncology
  - Radiation oncology
  - Surgery/surgical oncology
  - Pathology
  - Diagnostic radiology
  - Nursing
  - Others? ( social services, pharmacy, nuclear medicine, genetics, dentistry, nutrition therapy, physical/occupational therapy, pastoral care, pain/palliative care, mental health, clinical trials, and data management representatives; and fellows, residents, and other health care students)
- Does the referring physician attend the MCC?
- What is the role of the referring physician in the conference?
- Is attendance recorded at each meeting ? Are continuing professional development credits issued for attendance?
- Who presents the case?
- What clinical data is presented? Is there a standard format in which the information is presented?
- How is the meeting documented?
- Does your MCC use teleconferencing or videoconferencing? If so, under what circumstances would you use this format? [‘virtual MCC’]

1. Wenger E, McDermott R, Snyder W. Cultivating communities of practice: A guide to managing knowledge. Boston, MA: Harvard Business School Press; 2002. [↑](#footnote-ref-2)
2. Cancer Care Ontario – Communities of Practice. https://www.cancercare.on.ca/common/pages/UserFile.aspx? fileId=88179 [↑](#footnote-ref-3)
